# BOLD Correlates of Trial-by-Trial Reaction Time Variability in Gray and White Matter: A Multi-Study fMRI Analysis

**DOI:** 10.1371/journal.pone.0004257

**Published:** 2009-01-23

**Authors:** Tal Yarkoni, Deanna M. Barch, Jeremy R. Gray, Thomas E. Conturo, Todd S. Braver

**Affiliations:** 1 Department of Psychology, Washington University, Saint Louis, Missouri, United States of America; 2 Department of Radiology, Washington University School of Medicine, Saint Louis, Missouri, United States of America; 3 Department of Psychology, Yale University, New Haven, Connecticut, United States of America; James Cook University, Australia

## Abstract

**Background:**

Reaction time (RT) is one of the most widely used measures of performance in experimental psychology, yet relatively few fMRI studies have included trial-by-trial differences in RT as a predictor variable in their analyses. Using a multi-study approach, we investigated whether there are brain regions that show a general relationship between trial-by-trial RT variability and activation across a range of cognitive tasks.

**Methodology/Principal Findings:**

The relation between trial-by-trial differences in RT and brain activation was modeled in five different fMRI datasets spanning a range of experimental tasks and stimulus modalities. Three main findings were identified. First, in a widely distributed set of gray and white matter regions, activation was delayed on trials with long RTs relative to short RTs, suggesting delayed initiation of underlying physiological processes. Second, in lateral and medial frontal regions, activation showed a “time-on-task” effect, increasing linearly as a function of RT. Finally, RT variability reliably modulated the BOLD signal not only in gray matter but also in diffuse regions of white matter.

**Conclusions/Significance:**

The results highlight the importance of modeling trial-by-trial RT in fMRI analyses and raise the possibility that RT variability may provide a powerful probe for investigating the previously elusive white matter BOLD signal.

## Introduction

Reaction time (RT) is one of the most widely used measures of performance in experimental psychology. Many influential experimental paradigms (e.g., the Stroop task) employ RT as their primary dependent variable, and countless others measure RT in order to ensure that differences in response accuracy are not confounded with strategic shifts in response speed (the “speed-accuracy tradeoff”). Surprisingly, however, the analysis of RT has received limited attention in the functional neuroimaging literature (e.g., [1]–[4]). Although an ever-growing number of studies include RT as a trial-by-trial regressor in their analyses [e.g.], [ 1], [5], [6]–[9], such studies still represent a small fraction of the literature as a whole (for a quantitative review, see [3]). Moreover, the RT regressor is typically not the regressor of interest in such cases, but is included to ensure that activity differences between experimental conditions are not confounded by corresponding differences in RT. Finally, even in studies for which BOLD signal correlates of RT variability have been a focus of interest [e.g.], [1], [4,10], analyses have been conducted within relatively narrow, task-specific contexts.To our knowledge, no study has investigated the association between RT and brain activation across multiple experimental paradigms in order to identify potential task-general relations.

**Figure 1:**
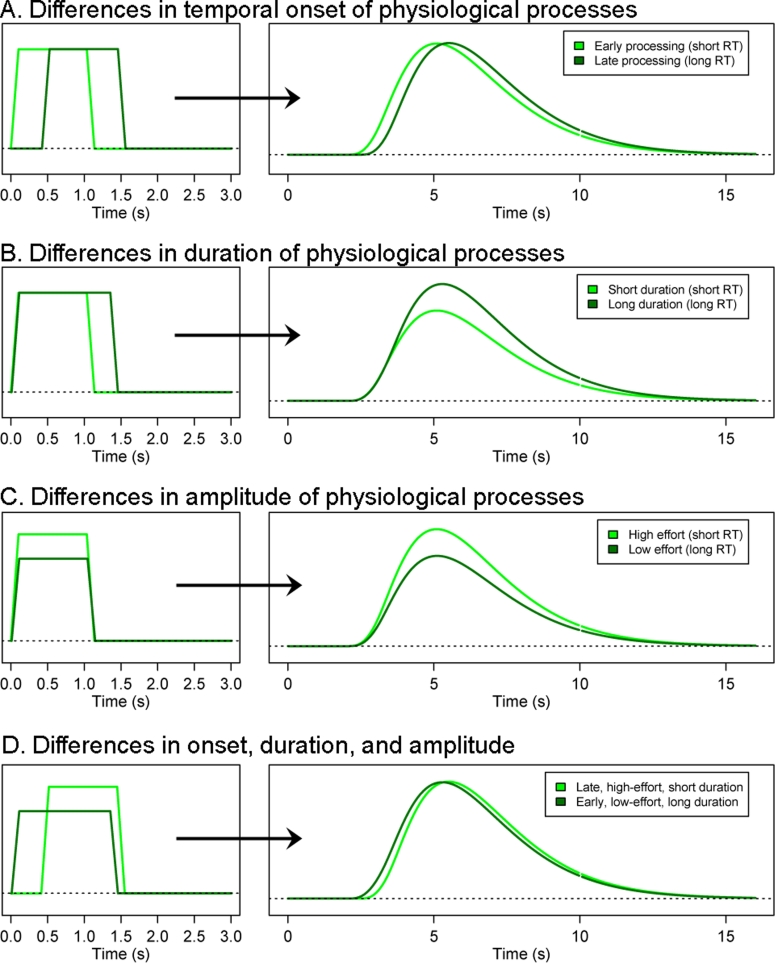
Hypothetical effects of changes in RT-related physiological processes on the BOLD response. (A) changes in onset. (B) changes in duration. (C) changes in amplitude.

There are several reasons to predict the existence of task-independent relations between activation and RT. First, many cognitive processes are expected to be time-locked to participants' overt responses (e.g., initiation of the motor response, processing of tactile or visual feedback, etc.). Consequently, the temporal onset of the hemodynamic response (HDR) should vary as a function of RT in sensorimotor brain regions; on trials when participants respond more slowly, activation should initiate later than on trials when participants respond quickly (Figure 1A). This prediction has been confirmed in a number of previous fMRI studies [2], [11] and serves as an important validation tool in the present context, because if a basic relation between RT and delayed HDR onset cannot be replicated across multiple studies, other kinds of relations are unlikely to be uncovered.

A second reason to predict a broad RT-brain activation relationship follows from the empirical observation that the BOLD signal measured by fMRI sums approximately linearly as a function of stimulation duration and intensity at short intervals [12]. If trial-by-trial differences in RT are viewed as naturally-occurring analogues of experimentally-manipulated differences in stimulus parameters, variation in either the amplitude or the duration of neurocognitive processes might be expected to reliably modulate RT. In cases where short-RT and long-RT trials are differentiated only by the *duration* over which some neurocognitive process unfolds, with no difference in amplitude, linear summation predicts that the BOLD response should attain a larger amplitude for trials with longer RTs (Figure 1B). For example, if one supposes that participants generally sustain attention to an on-screen stimulus until a relevant response is made, activation in brain regions that support attention (e.g., lateral frontal cortex and posterior parietal cortex [13], [14]) should increase approximately linearly with RT, other things being equal [cf. 8]. A number of previous fMRI studies have observed such positive relations in isolated paradigms [3], [5], [8], [15]–[17].

Third, one might predict that trial-by-trial differences in RT would be associated with changes in the amplitude or intensity of some cognitive processes rather than—or in addition to—their duration. For example, it is intuitive to think that natural fluctuations in cognitive effort or preparation level should produce trial-by-trial differences in RT. Other things being equal (i.e., assuming all trials have equal difficulty), as the cognitive resources allocated to a trial increase, the amplitude of activation in brain regions that support those resources should increase while RTs should decrease (Figure 1C). Thus, this view predicts that there should be a *negative* correlation between RT and the BOLD response in regions associated with deployment of task-related resources. Such a relation is particularly likely to emerge early on or even prior to each trial, when there is an opportunity to exercise preparatory processing [1], [18]. Limited evidence for such a relationship has been observed in a number of studies [1], [19]–[21]; for example, Weissman and colleagues recently found that decreased activation in frontoparietal regions just prior to trial onset was associated with longer RTs, a result they attributed to momentary lapses of attention [1]. However, the generalizability of such findings has not yet been systematically investigated.

These three possibilities (a temporal shift, a positive correlation with duration, and a negative correlation with amplitude) are not exhaustive, nor are they mutually exclusive. To the contrary, it is likely that RTs on most trials reflects a mix of influences, resulting in complex response shapes. Figure 1D illustrates the hypothetical response for two trials that differ in their onset, amplitude, *and* duration. The presence of multiple influences could potentially make RT-related activation difficult to detect if general trade-offs exist (e.g., if an increase in amplitude is precisely offset by a decrease in duration, it would be difficult to detect a difference in the resulting HDRs at short durations; Figure 1D). On the other hand, some types of influences might be stronger than others, resulting in easily-detectable RT effects across a variety of task paradigms. Ultimately, the question is an empirical one.

To test for the presence of systematic relations between RT and brain activation, the present study sought to assess the relationship between trial-by-trial variation in RT and brain activation at a relatively broad, task-independent level. Data from five different fMRI experiments were reanalyzed, with datasets chosen to span a range of experimental tasks (working memory, episodic memory, decision-making, and affective rating tasks), fMRI designs (event-related and mixed blocked/event-related), and stimulus modalities (words, affective pictures, faces, and numbers). We searched for regions that showed a consistent relationship across studies between BOLD activation and RT. Results provided strong evidence for two of three patterns predicted above. Specifically, we identified (a) temporal shifts in the onset of the BOLD response on trials with longer RTs throughout much of the brain, and (b) positive correlations between RT and activation in a number of frontal, parietal, and thalamic regions. Surprisingly, in addition to these expected RT effects in gray matter regions, we identified remarkably consistent relations between trial-by-trial changes in RT and activation strength in white matter regions, providing the most convincing evidence to date that it is possible to detect BOLD signal in white matter.

## Materials and Methods

### Datasets

We reanalyzed data from 5 previous experiments. Detailed methods for most studies have been previously reported, and we therefore summarize only key aspects of each study's methodology here (Table 1). All experiments were approved by the Washington University in St. Louis institutional review board.

**Table 1:** Key characteristics of the five datasets.

Sample	Population	n	Task(s)	Stimuli	Scanner	fMRI design	Mean RT (ms)
1	Young adults	50	3-back WM	W, F	1.5T	Mixed	994
2	Young adults	102	3-back WM	W, F	3T	Mixed	1066
3	Young adults	26	decision-making	N	3T	Mixed	642
4	Young adults	35	emotion ratings	P, W, F	3T	Event-related	1274
5	Young adults	39	memory	W, F	1.5T	Event-related	974

*Samples 1 and 2* were drawn from two large studies (n = 102 and 50, respectively) of healthy young adults who performed a 3-back working memory task involving face and word stimuli during scanning at 1.5 or 3 Tesla. A mixed blocked/event-related design [22] was used in both studies. Detailed methods have been previously reported [10], [23], [24]. *Sample 3* (n = 26) was drawn from a decision-making study involving a sample of healthy young adults. Participants selected cards from one of two decks and were rewarded with variable point rewards exchanged for money at the end of the experiment [25], [26]. A mixed blocked/event-related design was used to analyze the data. *Sample 4* was drawn from a larger experimental dataset investigating emotional processing in schizophrenia. For present purposes, only data from healthy control participants (n = 35) were analyzed. Participants rated the valence and arousal of affective stimuli (pictures, words, and faces) during scanning. A rapid event-related design was used to analyze the data. *Sample 5* was drawn from a larger experimental dataset investigating cognitive function in schizophrenia [27], [28]. Only data from healthy control participants (n = 39) were used. Participants were scanned while they performed several different memory encoding and working memory tasks involving word and face stimuli. Data was analyzed with a rapid event-related design. In total, the 5 samples comprised a sample size of n = 252. Responses in all samples were made manually by pressing a button, and RT was defined as the total time elapsed (in milliseconds) between the onset of a stimulus and registration of the participant's manual response.

### Data analysis

All analyses were conducted using a general linear model approach [GLM; [29]]. To identify the neural correlates of trial-by-trial differences in RT, we computed a new general linear model for each subject in each dataset, adding parametric regressors coding for RT on each trial. In principle, a parametric RT effect can be modeled in the GLM using a number of approaches. The most common approach is to use a variable impulse model, which models RT differences by varying the amplitude of the RT regressor across trials while holding its duration constant (for review, see [3]). However, Grinband and colleagues recently demonstrated that this approach incurs considerable power loss when the underlying signal varies only in duration and not in amplitude [3]. Grinband et al advocated the use of a variable epoch model that models RT by varying the duration and not the amplitude of the RT regressor, on the assumption that this approach more closely reflects the dynamics of underlying physiological processes. However, as noted above, it is theoretically possible for differences in RT to reflect differences in both the amplitude and the duration of underlying neurocognitive processes (e.g., when an increase in effort leads to a reduction in processing time; Figure 1D). To avoid making any assumptions about the shape of the RT-related response, we used an empirical estimation approach. In each data set, a Finite Impulse Response (FIR) basis set was used to estimate the influence of RT variability independently at 7 discrete time points following stimulus onset. This approach allowed us to accurately characterize the shape of the RT-related response at the cost of a slight reduction in detection power (due to consumption of additional degrees of freedom).

To increase power to detect RT-related activation, we employed the simplest design matrix possible in each sample. Thus, we collapsed across all non-essential experimental variables in each case, and estimated the influence of RT across all available trials. For example, the data for Samples 1 and 2 have previously been modeled using separate effects for different stimulus types, trial types, and/or mood induction conditions [10], [23], [30]. In the present analyses, we collapsed across such variables and modeled them all as a single effect coding for the difference between experimental trials and the fixation baseline. However, to ensure that putative RT effects could not be accounted for by other intercorrelated experimental variables (e.g., response accuracy), a subsequent validation analysis that included a broad range of experimental covariates was conducted in the largest sample (Sample 2). Additionally, because three of the samples used mixed blocked/event-related designs (samples 1–3), which require separate estimation of blocked and event-related effects [22], samples 1–3 retained separate effects for these two different estimates (in addition to RT).

For each subject within each dataset, RT values were standardized across trials prior to GLM estimation (i.e., each RT value was demeaned and divided by the standard deviation). The resulting standardized RTs were modeled independently at each of the 7 time points post-stimulus onset. No transformation was applied to the RT values before or after standardization. Thus, estimates of RT-related activation reflected a linear effect of RT. Volumes lacking an associated RT value (i.e., those occurring within baseline periods, or on trials in which subjects failed to respond) were assigned the mean standardized value of 0. This procedure ensured that response-less volumes would be assigned no weight in the regression and therefore would not influence the resulting first level (i.e., within-subject) estimates.

For each dataset, subjects' data were smoothed with a 3 mm FWHM smoothing kernel prior to GLM estimation. Regions that showed a significant relation with trial-by-trial RT variability were then identified by performing a whole-brain mixed-effects repeated measures ANOVA, with time (i.e., the 7 FIR regressors) as a fixed variable and subject as a random variable. The resulting F-test map representing the main effect of time was corrected for non-sphericity (i.e., autocorrelation in each participant's time series) and transformed to a z-map in order to weight samples of different sizes equally. Each map was statistically corrected for multiple comparisons using a voxel-wise (intensity) threshold of p<.001 and a cluster-wise (extent) threshold of 8 voxels. To assess the cross-experiment consistency of RT effects, we employed a conjunction analysis [31]; only clusters that showed a significant RT effect in all five samples were considered significant. Note that this approach is extremely conservative, because any region that failed to show an RT effect in at least one sample would be excluded from further consideration even if strong effects were observed in all other samples. Because the resulting map contained several very large clusters (>10,000 mm^3^) that each contained multiple anatomical structures, an automated peak-search algorithm was used to delineate boundaries of smaller ROIs by defining spherical ROIs around all peaks and repeatedly consolidating peaks within 20 mm of each other [32].

Although an ANOVA approach provides a powerful omnibus test for detecting RT-related brain activation that varies over time, a significant result implies only that there is *some* difference in activation over time, and provides no insight into the specific nature of that effect. We therefore conducted post-hoc analyses in order to characterize the pattern of activation present in the regions identified by the ANOVA analysis. Two linear contrasts designed to detect patterns of a priori interest were applied. First, to identify regions that showed a linear increase or decrease in response amplitude as a function of RT (Figure 1B–C), the coefficients of the 7 FIR regressors were weighted to fit a gamma hemodynamic response function (HRF [33]). Second, to identify regions in which underlying RT-related processes varied only in temporal onset and not in magnitude or duration (Figure 1A), the coefficients of the 7 FIR regressors were fit to a temporal derivative of a canonical HRF. The temporal derivative is formally equivalent to the difference between two identical HRFs staggered in time; thus, this contrast was optimized to detect regions in which activation “shifted” as a function of RT but did not otherwise change. Each of the two contrasts was applied at a regional level by testing the average of all voxels within each ROI identified by the ANOVA.

Finally, in addition to testing for specific response shapes, we conducted a more liberal exploratory analysis intended to identify any brain regions that showed RT-related activation in all five samples at *any* point in the activation time course. This analysis could potentially detect effects that were temporally consistent but not strong enough to attain significance in a full repeated-measures analysis in all five samples. At each acquisition timepoint (i.e., for each of the 7 FIR regressors), we identified voxels in which BOLD response magnitude was systematically correlated with trial-by-trial RT differences (p<.05 uncorrected, one-tailed) in the same direction in all five samples (i.e., positively correlated in all samples or negatively correlated in all samples). Note that although the HRF was modeled over seven acquisition volumes in all five samples, the length of each TR (or MRI repetition time) differed across studies, ranging from 2.36 s (Samples 1 and 2) to 3 s (Sample 4). This difference made the analysis more conservative, because it forced voxels to display consistent RT-related activation over a longer duration of time in order to be identified (e.g., an effect of RT at the fourth TR in all samples corresponded to a temporal window between 7–12 seconds post trial onset). We deemed this approach preferable to the less conservative and less computationally efficient method of sub-sampling TRs or interpolating time courses across multiple samples on a voxel-wise basis.

## Results

### Consistent RT-related activation in gray and white matter regions

**Figure 2:**
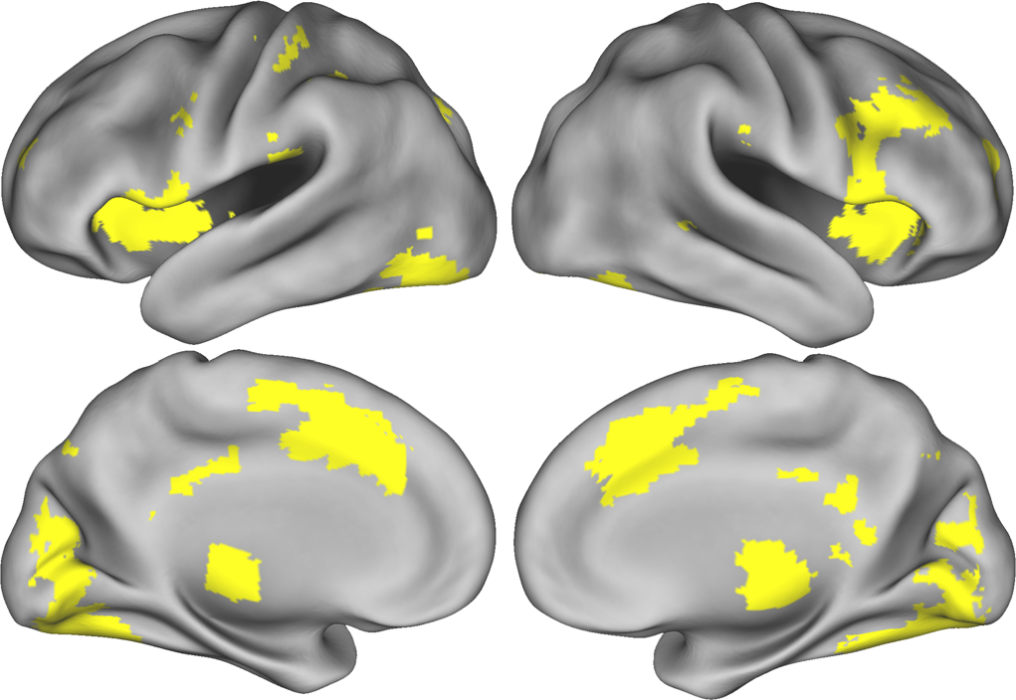
Cortical regions that showed significant RT-related activation in all five samples. Clockwise from top left: ,left lateral, right lateral, left medial, and right medial views of the cortical surface.

An initial whole-brain repeated-measures ANOVA identified all regions in which trial-by-trial RT variability correlated with BOLD signal changes in all five datasets. The resulting set of regions included large bilateral foci in medial frontal cortex, frontal operculum, lateral PFC, anterior PFC, visual cortex, medial cerebellum, and thalamus, as well as lateralized and/or more circumscribed foci in the precuneus, posterior cingulate cortex, and inferior parietal cortex (Figure 2; Table 2). Unexpectedly, in addition to these activations in cortical and subcortical gray matter regions, a number of activations were found in regions located within white matter. Specifically, RT-related activation was identified in the right lateral genu of the corpus callosum and in parts of the posterior corona radiata bilaterally (Table 2). The latter finding was surprising given that the BOLD signal in white matter is widely assumed to be considerably weaker in white matter than gray matter, presumably due to the lower metabolic activity of white matter [e.g., see 34]. To address potential sources of artifact that might have generated spurious RT-related signals in gray and/or white matter, we conducted several validation analyses that are reported later in this section.

**Table 2:** 

Region ID	Description	Hem.	BA	x	y	z	mm^3^
	***Frontal regions***						
1	Medial frontal cortex	M	6/8/32	1	12	48	14634
2	Medial frontal gyrus	M	6/24	−5	−6	53	4347
3	Anterior insula	L	13	−32	19	5	4509
4	Anterior PFC	L	10	−29	48	21	918
5	Precentral gyrus	L	6	−51	2	32	891
6	Ventrolateral PFC/anterior insula	R	45/44/13	41	22	3	11178
7	Dorsolateral PFC	R	9/46	44	12	32	6561
8	Anterior PFC	R	10	29	49	18	1215
	***Parietal/posterior cingulate regions***						
9	Posterior cingulate	M	31	0	−35	27	1593
10	Posterior cingulate	M	29	3	−45	6	1404
11	Precuneus	M	7	1	−76	36	891
12	Precuneus	L	19/7	−24	−75	33	270
13	Inferior parietal lobule	L	40	−41	−42	39	405
14	Postcentral gyrus	L	3/40	−41	−30	49	1701
	***Temporal/insular regions***						
15	Posterior insula	L	13/40	−51	−30	18	1026
16	Insula	L	13	−39	−2	5	5130
17	Postcentral gyrus	R	40	56	−24	22	324
18	Middle temporal gyrus	R	22	51	−44	−1	486
	***Visual/cerebellar regions***						
19	Cuneus	M	18	3	−81	29	3456
20	Cerebellum/visual cortex	M	17/18	6	−68	−27	16443
21	Fusiform gyrus	L	19	−41	−68	−9	7209
22	Lingual gyrus	L	18/17	−12	−62	1	9369
23	Cerebellar tonsil	L		−37	−61	−32	14499
24	Culmen (cerebellum)	R		27	−53	−22	15336
	***Subcortical regions***						
25	Midbrain	M		−1	−27	−8	1458
26	Thalamus	L		−21	−20	4	6291
27	Thalamus	R		12	−18	8	4428
	***White matter regions***						
28	White matter	L		−22	19	19	216
29	White matter	L		−24	−35	22	2025
30	White matter	R		−33	−39	−1	405
31	White matter	R		19	26	10	486
32	White matter	R		25	−45	21	594
33	White matter	R		20	−5	28	729

**Figure 3:**
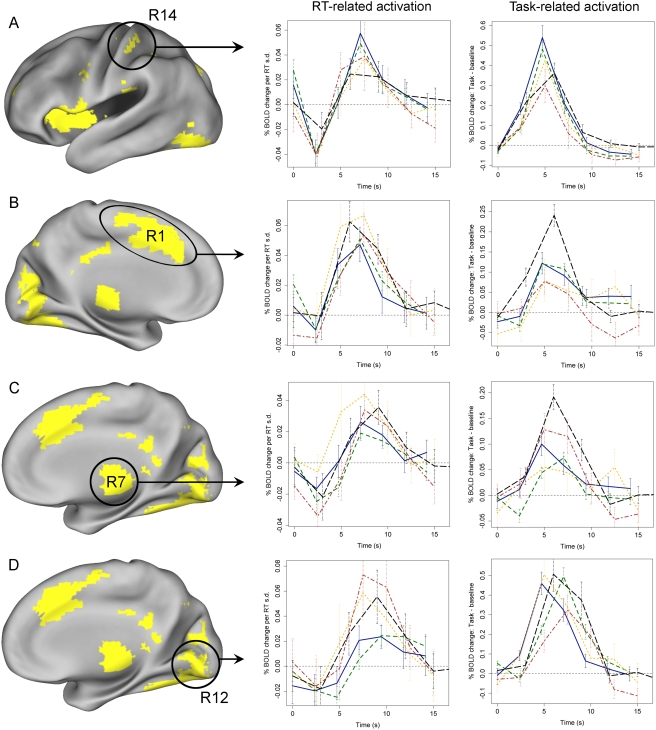
Time courses of RT-related activation in representative gray matter ROIs. Each line represents activation in a different sample. Left time course column: RT-related activation; right time course column: general task-related activation (i.e., task vs. baseline). Region labels (R14, R1, R7, R12) refer to region IDs in Table 2. Error bars reflect 95% confidence intervals.

**Figure 4 pone-0004257-g004:**
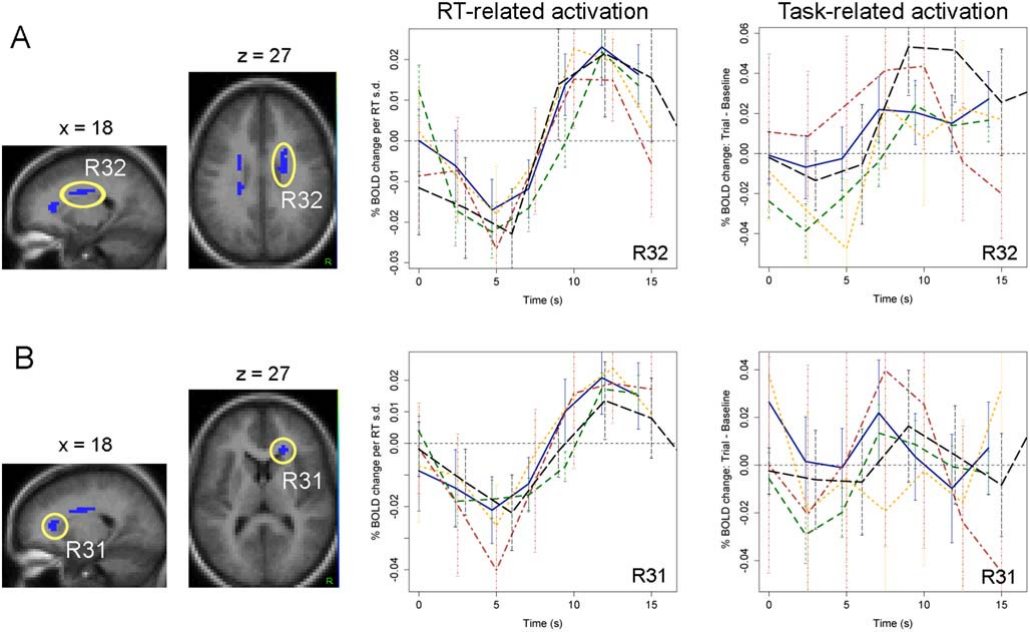
Time courses of RT-related activation in representative white matter ROIs.

**Figure 5:**
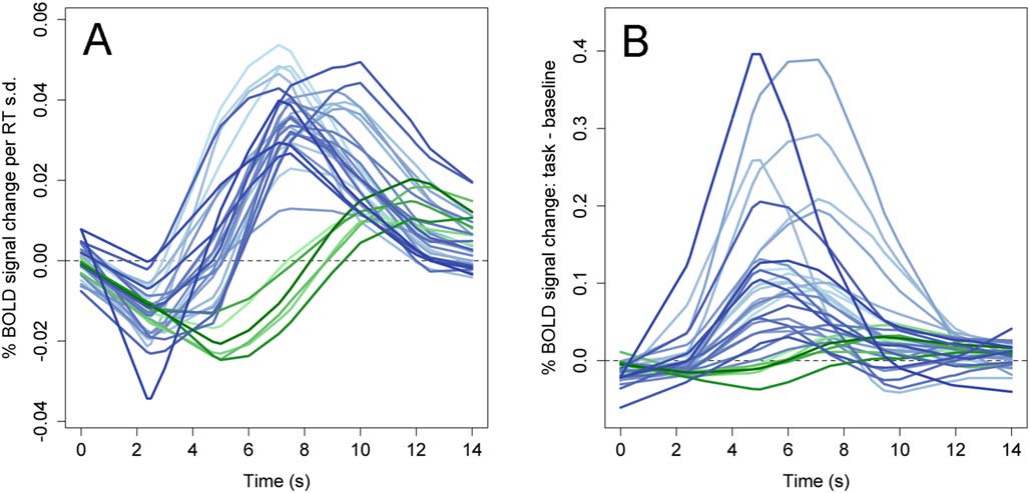
Mean RT and task-related time courses in all ANOVA ROIs. Each time course represents the time course of RT-related activation (A) or task-related activation (B) in a single region, averaged over all five samples. Blue: gray matter ROIs; green: white matter ROIs.

To characterize the pattern of RT-related activation within the regions identified by the whole-brain ANOVA, we employed two approaches. First, we visually inspected the empirically estimated time course of RT-related activation in each ANOVA ROI. RT-related timecourses for each of the five samples are presented for several representative gray matter (Figure 3) and white matter (Figure 4) regions. Figure 5A displays the mean timecourse averaged over all samples for each of the 33 ANOVA ROIs. The time courses illustrate three important points. First, RT-related activation showed a marked degree of spatiotemporal consistency. The shape of the response generally differed to a greater extent across brain regions within a single sample than across samples within a single region (Figures 3–4). Thus, regional differences in the shape of the HDR appear to manifest reliably not only in standard experimental contrasts [35], [36] but also with respect to functional differences in RT. Second, virtually all gray matter regions showed both (a) an initial “dip” in the RT-related time course approximately 2s post-onset, and (b) uniformly greater activation for longer RTs thereafter (Figure 5A). This pattern is consistent with the presence of both a temporal shift in the response (i.e., later initiation of the response for longer RTs) and a time-on-task effect (i.e., greater summation of the BOLD response on long-RT trials due to increased processing duration). Finally, strikingly different response shapes were observed in gray and white matter regions, with the latter exhibiting a smaller amplitude and a substantial delay in time-to-peak (approximately 10–12 s versus 7–10 s) relative to the former (Figure 5A).

**Figure 6:**
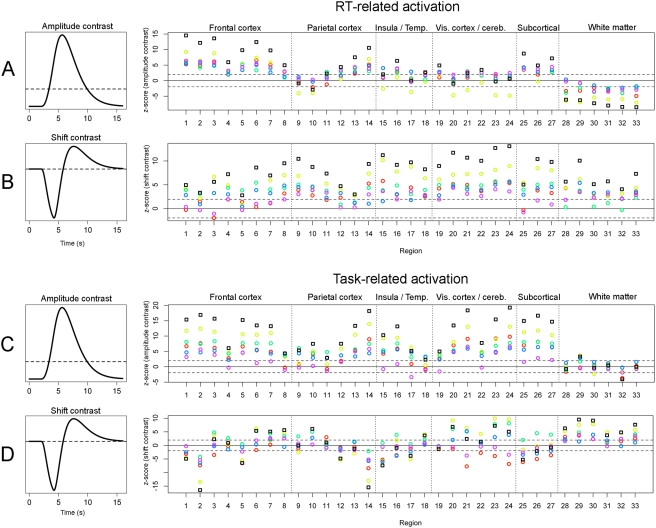
Statistical fit of RT-related and task-related activation to a priori linear contrasts. Top (panels A–B): RT-related activation. Bottom (C–D): task-related activation. Contrast weights for the amplitude contrast (panels A and C) and temporal shift contrast (panels B and D) are displayed on the left. The statistical significance (z-score) of the resulting test is displayed on the right for each of the 5 samples in each of the 33 ANOVA ROIs. Each color represents a different sample; black squares represent the fixed-effects z-score sum of all studies (see text). Region numbers correspond to IDs in Table 2 and Figures 3–5. Dashed lines represent a p<.05 cut-off (|z| = 1.96).

Second, for each ANOVA ROI, we applied linear contrasts designed to identify activation that showed either (a) a temporal shift in the hemodynamic response without a corresponding change in magnitude (*shift contrast*); or (b) a linear increase or decrease in the magnitude of activation as a function of RT (*amplitude contrast*). Figure 6 (A, B) displays the results of these two contrasts for each sample in each ROI. Each colored circle represents the z-score obtained in a different sample. The black squares represent the fixed-effects sum of all five z-scores (i.e., the sum of all z-scores divided by the square root of the number of studies [37]). The Figure supports several conclusions. First, consistent with the above qualitative interpretation of Figure 5A, all 33 ROIs showed a positive temporal shift (i.e., a later peak for longer RTs; all *p*s<.05), consistent with the notion that a general delay in the initiation of task-related processing is one contributor to longer RTs. Shift effects were particularly robust in visual, cerebellar, and parietal regions that are presumably involved in processing sensory feedback related to the motor response.

Second, highly significant positive correlations between RT and activation were found predominantly in frontal regions, though several regions in parietal cortex and the thalamus also showed a positive correlation (p<.05). Negative correlations between RT and activation were found only in the 6 white matter ROIs. However, as noted above, the HDR in white matter ROIs appeared to evolve much more slowly than the model HDR. Thus, the apparent presence of negative correlations with RT may reflect a failure of the model-based amplitude contrast to accurately characterize the shape of the white matter response. Visually, time courses of RT-related activation in white matter ROIs appeared in large part to reflect delayed onset for longer RTs rather than a change in amplitude (Figures 4,5). The divergence between model-based and inspection-based interpretations of RT-related activations underscores the value of empirically estimating RT-related time courses rather than using a strictly model-based approach.

### Relation between RT-related and task-related activation

Because RT-related changes in activation were statistically orthogonal to more general differences in task-related activation (i.e., the contrast between task performance and a fixation baseline), we next investigated the relation between these two types of effects. For each ROI that showed an effect of RT, we estimated and plotted the corresponding task-related responses (Figures 3–5) and applied the same linear contrasts testing for shift versus amplitude differences (Figure 6C,D). Task-related responses differed qualitatively from RT-related responses in both gray and white matter ROIs. In gray matter ROIs, task-related changes in the amplitude of activation were generally stronger than corresponding RT-related effects. That is, z-scores for the amplitude contrast were consistently larger for task-related effects than for RT-related effects, despite the fact that it was the RT effect that was used to define the ROIs in the first place (compare z-scores in Figure 6, panels A vs. C). In contrast, in white matter ROIs, a striking discrepancy was observed between RT-related and task-related responses. Task-related responses were much less reliable than RT-related responses, showing little consistency across studies and generally failing to resemble a canonical HRF (Figures 4–5). This divergence is consistent with previous failures to detect a reliable BOLD signal in white matter using conventional subtractive contrasts, and suggests that it is specifically the RT-related modulation of the BOLD signal in white matter that is strong enough to be reliably detected.

### Exploratory analysis

**Figure 7:**
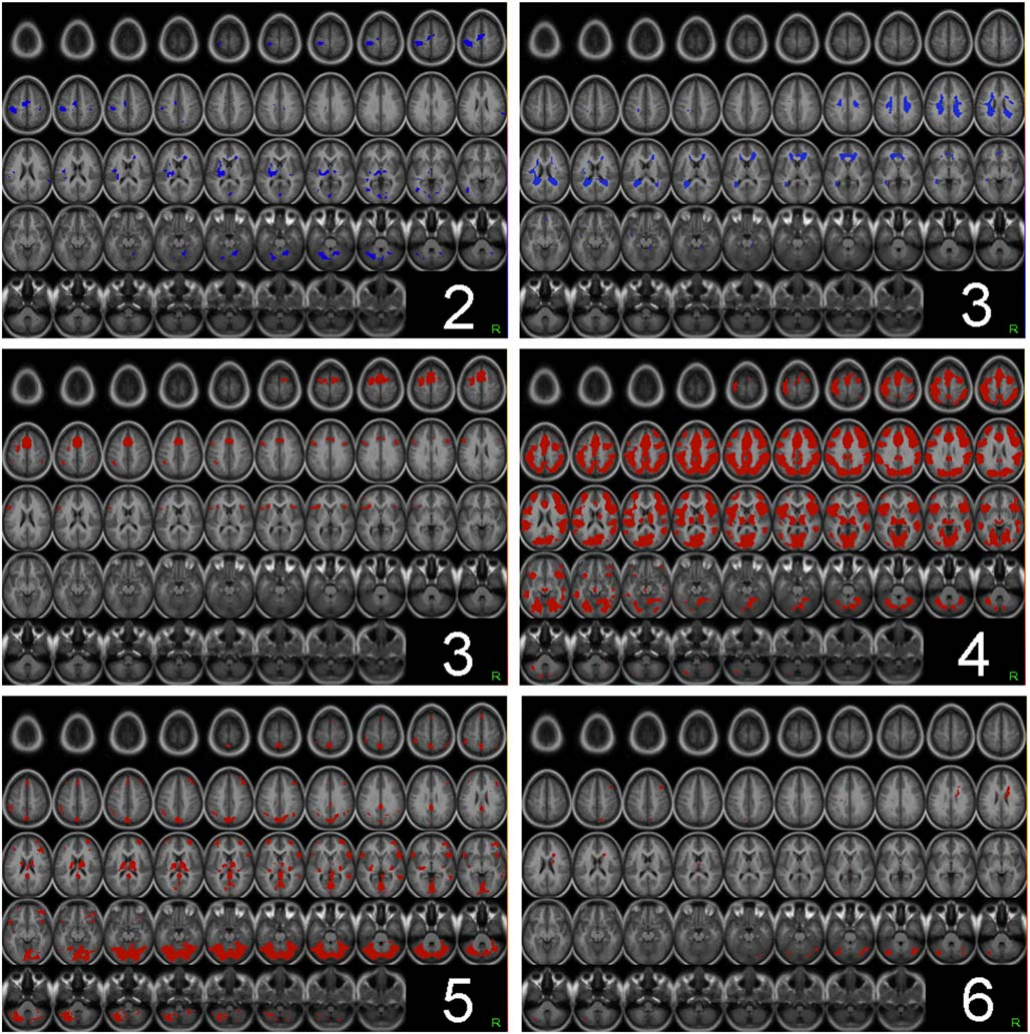
Timepoint-specific negative and positive correlations with RT. The white digit in each panel indicates the timepoint (i.e., the acquisition volume relative to trial onset) at which the correlation with RT occurred. Blue: negative correlations with RT in all five samples; red: positive correlations with RT in all five samples.

To complement the whole-brain ANOVA, which identified regions that showed a highly significant effect of RT across the entire time course (i.e., a main effect of time), we conducted a more liberal exploratory analysis intended to identify any brain regions that showed consistent RT-related activation across studies at specific points in the activation time course. A separate search was conducted at each acquisition volume for regions that correlated with RT in the same direction in all samples. Results were broadly consistent with the ANOVA results (Figure 7). At TR 1 (0–3 seconds post-onset), no region correlated either positively or negatively with RT. At TR 2 (2.36–6 seconds post-onset), no positive correlations with RT were found, but negative correlations with RT were observed in somatosensory cortex, mid-cingulate gyrus, thalamus and cerebellar cortical regions. At TR 3 (4.72–9 seconds), positive correlations with RT were found in medial frontal cortex, lateral prefrontal cortex, and frontal operculum. Negative correlations were found diffusely in white matter. Thereafter, at TRs 4–6 (7.08–18 seconds post-stimulus onset), correlations with RT were exclusively positive, and were observed throughout much of the cortex, basal ganglia, thalamus, and cerebellum. No correlations with RT were detected at TR 7.

### Validation analyses

The presence of consistent correlations between activation and RT across fMRI datasets involving different samples, scanners, experimental tasks, and analysis streams suggested that the association was unlikely to depend on task- or study-specific factors, e.g., stimulus modality or length of response window. However, RT could still be confounded with other task-general experimental factors such as response accuracy or trial difficulty, or with systematic artifact sources such as head movement. To assess the impact of such factors, we conducted a series of validation analyses in the largest sample (sample 2, n = 102). First, we created new GLMs that included additional regressors for several experimental covariates (for full details of the experimental design, see [24]). These included (a) response accuracy (error vs. correct), (b) stimulus type (words vs. faces), (c) emotion condition (approach, neutral, and withdrawal), and (d) 3-back trial type (lure, target, and novel). The effect of RT remained highly significant in all 33 ROIs (all *F*s>6.4, *p*s<.0001). Note that this analysis is highly conservative, as it removes *any* variance shared between RT and other variables (e.g., accuracy), regardless of which variable has causal primacy.

**Figure 8:**
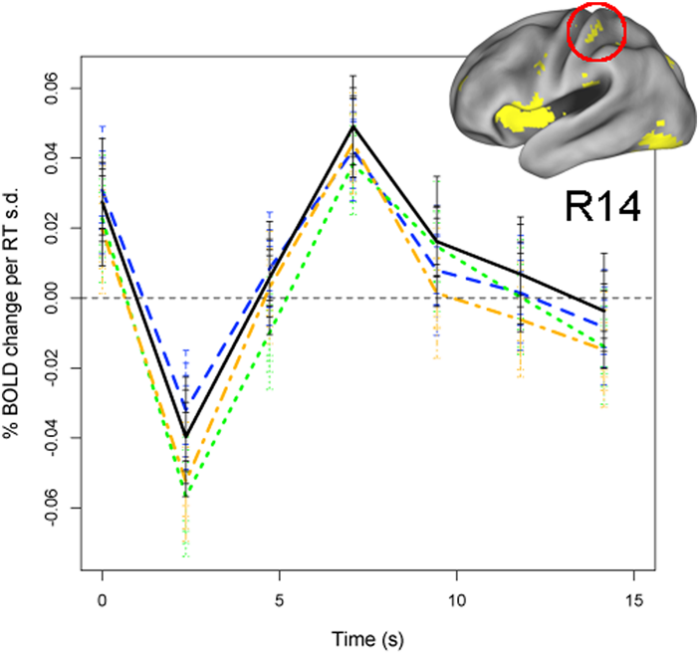
RT-related activation in somatosensory cortex estimated separately by trial type in Sample 2. Each colored line represents the time course of RT-related activation estimated for a different trial type, after controlling for a range of experimental covariates (see text). The black line represents the original estimate (cf. Figure 4A) when collapsing across all trial types. Error bars indicate 95% C.I.

Second, we recomputed the above model with RT estimated separately for each of the three types of 3-back trial types (lure, target, and novel) in order to determine whether the relation between RT and brain activation held not only at the overall task level but also for different experimental conditions associated with different cognitive demands [38]. This analysis was even more conservative than the previous analysis, because each of the covariates (stimulus type, emotion condition, response accuracy, etc.) was also estimated separately for each trial type in order to ensure consistent treatment of RT, effective tripling the degrees of freedom consumed. Nonetheless, despite the substantial reduction in power, the RT effect remained significant in all 33 ROIs for target trials (p<.05), in 32/33 ROIs for novel trials (p<.05), and in 23/33 ROIs for lure trials (p<.05; note that the reduction in number of significant effects for lure trials likely reflected decreased estimation power, because lure trials comprised only 16% of all trials). Critically, in regions that showed a significant RT effect for all 3 trial types, time courses were virtually indistinguishable in shape (e.g., Figure 8). Thus, the relation between RT and brain activation held not only at an overall task level but also for specific experimental conditions.

Third, we assessed the impact of head movement on estimates of RT-related activation. Although a six-parameter affine transform was used to correct for movement during preprocessing, it was conceivable that a residual influence might bias the GLM estimates if movement happened to be correlated with trial-by-trial variation in RT. This concern was particularly applicable to the observed white matter effects, because the intensity of the BOLD signal in white matter was weaker than in gray matter, and thus potentially more susceptible to systematic noise. To control for movement, we computed two separate sets of GLMs, each of which added several movement regressors to the existing set in each study. One set coded for directional movements using 12 separate regressors. Six regressors coded for absolute shift in head position relative to the start of the first run, and six regressors coded for volume-by-volume differences in movement. Of each set of six, three regressors coded for translation in the x, y, and z planes and three regressors coded for rotation in the same planes. The second set of GLMs coded for absolute rather than directional movement, and included two different regressors, one reflecting total translational movement and one reflecting total rotational movement (each computed as the square root of the sum of squares of x, y, and z movements in each volume). The RT estimate was not affected in either analysis. Effects remained significant across all ROIs in both models (p<.05 in one ROI; all *F*s>6, *p*s<.0001 in all other ROIs).

Fourth, we constructed a GLM that controlled for the serial position of each trial within the overall scan sequence (i.e., trial number). This analysis controlled for potential confounding influences of practice or fatigue effects. We reasoned that if RT varied systematically as a function of task experience (e.g., decreasing over time as responses became more automated, or increasing over time because of greater fatigue), and if for some reason there was a systematic change in BOLD signal in gray or white matter over the course of the experiment, one might expect a spurious correlation between activation and RT to emerge (note that this effect would have to be independent of scanner drift, which was modeled using nuisance regressors in all GLMs). However, no such effect was observed. When controlling for trial number, the RT effect remained highly significant in all ROIs (all *F*s>9.1, *p*s<.0001).

Finally, we systematically inspected the preprocessing stream used in all five samples in order to identify any potential steps that might introduce systematic artifact correlated with trial-by-trial RT differences. No obvious candidate emerged. The most obvious candidate step would be a correction for global intensity differences, which has previously been shown to induce spurious white matter deactivations [39] (i.e., if the average intensity of the entire volume changed as a function of RT due to changes in gray matter, normalizing all volumes to have the same mean could potentially induce a spurious shift in white matter signal). However, such a processing step was not used in any of the samples.

## Discussion

The primary finding of the present study was the identification of gray and white matter brain regions in which activation correlated systematically with trial-by-trial differences in RT across a broad range of experimental tasks. Strong evidence was found for both temporal shifts in RT-related activation, presumably reflecting delayed onset of cognitive processing, and uniform positive correlations between RT and activation in frontal regions, likely reflecting a “time-on-task” effect of sustained attention. Additionally, strong evidence emerged for a reliable effect of RT on BOLD signal in white matter. We now turn to discuss the theoretical and methodological implications of these findings.

### Time-on-task versus temporal shift effects of RT

Virtually all RT-related activations identified in the present study could be characterized as either an amplitude increase (i.e., systematically greater activation for long RTs than short RTs) or a temporal shift (i.e., delayed onset of the HDR for long RTs relative to short RTs with little or no change in shape). These two patterns showed a moderate degree of spatial segregation, with amplitude effects restricted primarily to frontoparietal and thalamic regions, whereas temporal shift effects were ubiquitous throughout the brain but were strongest in somatomotor, visual, cerebellar, and posterior midline cortical regions. This anatomical dissociation is consistent with a division of labor between brain regions that support cognitive processes that occur prior to the motor response and brain regions that support response-locked processes such as motor execution, tactile feedback processing, and processing of visual display changes.

The fact that positive correlations between RT and BOLD amplitude were found primarily in frontal regions is consistent with the conventional wisdom that MFC and lateral PFC regions are central components of a cognitive control network broadly implicated in supporting effortful, goal-directed activity [40], [41]. Of particular relevance is a recent multi-study analysis by Dosenbach and colleagues in which the authors identified highly consistent sustained task-related activations in MFC and frontal operculum regions that overlapped closely with those identified in the present study [42]. Dosenbach and colleagues suggested that these regions are necessary for the implementation and maintenance of a goal-directed task set. While they focused on temporally extended activation that persisted throughout entire task blocks, the present findings point to a direct analog at much shorter intervals. Given that participants are usually free to relax their attention and “mind wander” for the remainder of a trial once they have responded to the stimulus, neural activity in frontal regions necessary for sustaining goal-directed attention should persist for the duration over which attention is maintained [cf. 43], [44]—a duration closely indexed by RT. Because the BOLD response sums approximately linearly overt short intervals [12], trials with long RTs should then produce proportionally larger activations in the same frontal regions.

It is important to note that the presence of robust time-on-task effects does not conclusively rule out the possibility that there are other relatively broad relations between brain activation and RT variability. At very short intervals (e.g.,<2 seconds), changes in the duration versus amplitude of physiological processes are likely to exert similar effects on the BOLD response (e.g., compare panels B and C in Figure 1). If a general tradeoff exists between the amplitude of processes supported by frontal regions and their duration (e.g., if a 20% increase in frontal activation results in a 20% reduction in RT, other things being equal), it may be difficult if not impossible to detect using the present approach. Thus, the present findings should not be taken to imply that increases in preparatory processing or mental alertness (which presumably would be associated with increased frontal activation [20], [45]) have no effect on RT. Considerable evidence demonstrates the existence of such effects; for example, increased ACC and DLPFC activation predicts faster and more accurate responses during upcoming trials [18]–[21], [46]. What the present results do suggest is simply that the influence of *task-general* preparatory or alertness-related processes on RT is relatively negligible in comparison to the dominant time-on-task effect. This conclusion is entirely compatible with reports of larger preparation-related decreases in RT in studies that involve specific kinds of experimental conditions (e.g., the presence of cue information), or with the general notion that variability in mental preparedness (e.g., the occurrence of attentional lapses prior to trial onset) has an influence on RT [1], [8]).

Interestingly, the present findings do provide some evidence for a weak effect of cognitive preparation or alertness on RT. Virtually all RT-related ROIs showed a small negative correlation with RT very early in the activation time course (Figure 5). Moreover, the early decreases contrasted sharply with task-related responses in the same regions, which were strictly positive-going in most cases. Weissman and colleagues [1] recently suggested that these early negative correlations with RT are functionally coupled to the later positive correlations. Specifically, they argued that deactivations in regions associated with attentional control reflect lapses of attention, and that the late positive increases reflect a subsequent attempt to compensate for such lapses by reasserting additional control. However, the present results argue against such an interpretation, because (a) in frontal regions associated with cognitive control, the late positive correlations with RT were substantially larger than the early negative correlations, and (b) an early dip in activation was observed in virtually all regions, including sensorimotor regions that are unlikely to play a role in asserting control. A more plausible interpretation is that the two phenomena are largely independent. That is, lapses of attention contribute to the ubiquitous temporal shifts we observed (i.e., task-related processing initiates slightly later in virtually all brain regions immediately following a lapse), whereas frontal regions play a preferential role in sustaining controlled processing for the duration of a trial until a response is made.

### Methodological implications of a time-on-task effect

The present findings have clear and important methodological implications for the inclusion (or lack thereof) of RT as a covariate in functional neuroimaging studies. It is both common sense and an axiom of experimental psychology that RT and accuracy are inversely related under most circumstances—that is, the longer a person takes to respond, the more likely their response is to be accurate, assuming that experimental conditions are held constant. In behavioral studies that use response accuracy as the primary dependent variable, it is standard practice to explicitly rule out the possibility of a speed-accuracy tradeoff, e.g., by statistically covarying out RT or demonstrating that there are no meaningful differences in RT between conditions. This concern is equally applicable to neuroimaging studies, where differences in activation between two conditions could theoretically be confounded with differences in both RT *and* response accuracy.

Surprisingly, while many fMRI researchers routinely take pains to eliminate response accuracy differences as a potential confound (e.g., by only analyzing trials with correct responses), relatively few studies have systematically controlled for trial-by-trial RT differences [e.g.], [5], [7,47]; a recent survey of 170 fMRI studies found that only 9% had explicitly modeled RT [3]. The present results suggest that this omission may not be benign. The strength of the RT effects we observed in frontal regions suggests that RT variability may explain a considerable amount of variance in frontal activation in most tasks. If two experimental conditions differ substantially in mean RT, a corresponding difference in frontal activation is likely to be observed *irrespective of any other differences in task structure*. Moreover, given that the present study focused only on RT-related activation that was relatively independent of task-specific demands, one might expect similar, but more task-specific, time-on-task effects to be present in other brain regions.

At present, there is no easy way to determine the extent to which quantitative differences in trial-by-trial RT variability might account for fMRI effects previously attributed to qualitative differences between experimental conditions. Relatively few studies have directly contrasted effects with and without RT covariates, and these studies have reported mixed results. In some cases, controlling for RT produces no discernible impact on experimental effects of interest [e.g.], [24], [47], [48,49]. In other cases, some effects of interest may be eliminated or even reversed when RT is explicitly modeled [e.g.], [7,50]. It is important to note that the widespread practice of including the temporal derivatives of modeled responses in GLM analyses in order to account for temporal differences in HDR onset will have virtually no influence on estimates of RT-related activation in regions that show a time-on-task effect. Including temporal derivatives in the GLMs used in the present study would likely have reduced or eliminated the temporal shift effects identified in somatomotor, visual, and cerebellar regions; however, regions that show relatively uniform positive activations as a function of RT (e.g., MFC and lateral PFC) would be largely unaffected, because activation in the latter regions appears to increase at virtually all timepoints. To account for such effects, trial-by-trial differences in RT should be explicitly modeled within the GLM—either by empirically estimating the RT-related response, as in the present study, or by using an alternative approach such as a variable impulse or variable epoch model (for discussion, see [3]).

Given that the interpretation of many results might change considerably depending on whether effects are independent of RT or not, there is a clear incentive for researchers to include RT as a covariate in analyses. A particularly informative approach might be to analyze one's data both with and without RT in the model, enabling more precise inferences about whether the neurocognitive processes recruited by different experimental conditions vary quantitatively or qualitatively. Some hypotheses might be confirmed by demonstrating that differences in frontoparietal activation are fully explained by RT differences, and are purely quantitative in nature; for example, one might hypothesize that increasing the load in a Sternberg working memory task [51] from 3 to 4 items should produce a strictly quantitative change in brain activation and RT, and that no difference in the former should remain after controlling for the latter. In contrast, other hypotheses might require a demonstration that activation differences remain significant even after controlling for RT. For example, one would expect activation differences for word naming versus non-word naming to remain significant even after controlling for RT, reflecting the fact that word naming can recruit pathways that non-word naming cannot [5]. In general, there is no reason, save perhaps expediency, *not* to include RT as a covariate in parallel fMRI analyses, while the potential benefits are considerable.

### Reliable effects of RT on BOLD signal in white matter

A surprising finding of the present study was the presence of a consistent association between trial-by-trial RT variability and BOLD signal in white matter regions. The precise nature of this association is somewhat unclear due to the atypical shape of the hemodynamic response in white matter (Figure 5)—the white matter response appears to have the same fundamental characteristics as the gray matter response, but evolves much more slowly. A parsimonious interpretation of the present findings is that RT effects in white matter regions reflect temporal shifts similar to those observed in gray matter regions such as somatosensory cortex that are simply “stretched” in time. That is, on trials with long RTs, the BOLD response in white matter is delayed relative to trials with short RTs, presumably because processes supported by white matter (e.g., conduction of action potentials along corticospinal pathways) initiate later in time. However, an alternative possibility is that the very late increase in RT-related activation observed in white matter reflects an “overshoot” phase of a negative-going impulse. On this view, increases in white matter activation might be systematically associated with shorter RTs because they serve some functional purpose, e.g., facilitating more rapid communication between different gray matter regions on trials with short RTs.

Interpretative issues aside, the identification of a reliable BOLD signal in white matter has potentially important implications for fMRI methodology and our understanding of the BOLD signal. It is widely assumed in the functional neuroimaging community that it is difficult if not impossible to reliably detect BOLD responses in white matter because metabolic rates, vascular density, and cerebral perfusion are much lower in white matter than in gray matter [34], [52]. Logothetis [53] captured this sentiment in a recent review of mechanisms underlying the BOLD signal, noting that “activation of the white matter has been rarely reported in the neuroimaging literature, and a reasonable investigator may doubt the presence of a BOLD signal in white matter altogether” (p. 755). While there is no doubt that the present findings are unexpected, there are several reasons to believe that the observed white matter activations veridically reflect underlying physiological processes.

First, it is worth noting that the widespread assumption that BOLD signal is undetectable in white matter is based largely on negative evidence—that is, a failure to observe significant white matter activations. There is no positive evidence to suggest that such activations are impossible in principle. To the contrary, there are good reasons to predict the presence of BOLD signal in white matter. The BOLD signal reflects a complex interplay between changes in cerebral blood flow (CBF), cerebral blood volume (CBV), and oxidative metabolism [53]–[55]. Such factors might be expected to operate in white matter as well as gray matter, because (a) the balance between oxidative metabolism and blood flow is similar in white and gray matter (as evidenced by similar oxygen extraction fractions in white matter [56], [57]), and (b) CBF and CBV are only 2–3 times lower in white matter than in gray matter [57]–[60]. Thus, in principle, white matter BOLD signal should be detectable given a sufficiently large sample size, sensitive acquisition techniques, and a sufficiently sensitive analytic probe. Moreover, recent discoveries that some types of glial cells participate in glutamatergic signaling [61], [62] and can even generate action potentials [63] provide potential theoretical bases for the presence of functional relationships between cognitive processes and BOLD signal in white matter.

Second, from a statistical standpoint, the probability of jointly observing consistent white matter activations in all five samples is infinitesimally small (p<.001^5^). Moreover, as illustrated in Figure 4, different samples produced extremely similar RT-related time courses in virtually all regions, despite the fact that the model-free ANOVA procedure used to identify ROIs imposed no constraint on the shape of activation in each case. Thus, while it is conceivable that white matter RT effects might reflect an unidentified confounding variable, they cannot be rejected as false positives.

Third, and related to the above concern about potential confounds, consistent white matter effects were observed in samples obtained using different fMRI scanners, experimental tasks, and analytic designs. Thus, any potential source of artifact would have to be extremely general. The most obvious candidate, namely, head movement, had no discernible influence on the RT effect when explicitly modeled in the GLM. Similarly, controlling for a variety of experimental factors (e.g., response accuracy, trial number, etc.) or modeling the RT effect separately for different types of trials did not affect the results.

Fourth, it is important to note that white matter effects were specific to the trial-by-trial RT effect in the present datasets. We found no consistent white matter activation across studies when contrasting task-related activation with baseline. Thus, the present results are entirely compatible with previous failures to detect a white matter BOLD signal. A plausible explanation for the fact that the RT-related signal appears to be much more reliable than the task-related signal is that the production of an overt motor response may require generation of highly synchronized and relatively strong impulses in corticospinal motor axons that are conveniently time-locked to the onset of the motor response. In contrast, when activation during two experimental conditions is contrasted subtractively (e.g., task vs. baseline), the BOLD signal in white matter is likely to reflect the noisy summation of many different impulses that vary in time and strength in both conditions (e.g., continuous communication between different cortical and subcortical regions is liable to occur during both task periods and fixation baseline), making significant differences much more difficult to detect.

Fifth, although reports of BOLD signal in white matter are rare, several studies have in fact observed such effects using experimental approaches broadly consistent with the present focus on RT variability. Two recent studies that used visual-manual RT tasks to investigate the neural correlates of interhemispheric transfer found greater activation in the corpus callosum on trials that required interhemispheric transfer of information than on trials that did not [64], [65]. Strikingly, both studies reported white matter activation in a region of the right genu of the corpus callosum (peak coordinates: 14, 28, 16 and 10, 26, −4, respectively) that overlapped closely with an ROI identified in all five samples in the present study (center-of-mass coordinates: 20, 26, 9).

Finally, several diffusion tensor imaging (DTI) studies have found correlations between individual differences in mean RTs and white matter integrity [66]–[69]. These correlations are universally negative, i.e., individuals with greater white matter integrity have shorter RTs across a range of different cognitive tasks. Although the individual differences results of DTI studies are not directly comparable with the within-subjects (i.e., trial-by-trial) BOLD effects identified in the present study, the DTI results nevertheless provide a conceptual corroboration of the present results inasmuch as they suggest that variability in white matter structure has functional implications for RT variability. Future studies could combine DTI and BOLD data to directly test for a relationship between the two measures. For example, one might predict that individuals with greater structural integrity in white matter tracts should have a larger dynamic range of activation, and might therefore show greater modulation of white matter BOLD as a function of trial-by-trial RT differences. In sum, while we remain open to the possibility that the white matter activations reported here will prove to be artifactual, we believe there are sufficient methodological and theoretical grounds to warrant further investigation.

### Conclusion

The present results provide strong support for the existence of task-independent relationships between trial-by-trial differences in RT and gray and white matter activation. The presence of robust time-on-task effects in frontoparietal brain regions underscores the importance of explicitly modeling RT in fMRI analyses, whether as a covariate of no interest or as a variable of interest in its own right. Although the association between white matter activation and trial-by-trial differences was not predicted *a priori,* and its precise nature remains unclear, the current study provides the strongest evidence to date that BOLD signal can be reliably detected in white matter. Future investigations could potentially use trial-by-trial changes in RT to probe the integrity of white matter function as well as the physiological basis of the BOLD signal.
